# A Proposed Framework to Describe Movement Variability within Sporting Tasks: A Scoping Review

**DOI:** 10.1186/s40798-022-00473-4

**Published:** 2022-06-27

**Authors:** Jake Cowin, Sophia Nimphius, James Fell, Peter Culhane, Matthew Schmidt

**Affiliations:** 1 School of Health Sciences, University of Tasmania, Newnham, TAS Australia; 2 Tasmanian Institute of Sport (Sports Performance Unit), Prospect, TAS Australia; 3 School of Medical and Health Sciences, Centre for Human Performance, Edith Cowan University, Joondalup, WA Australia; 4 School of Health Sciences, University of Tasmania, Hobart, TAS Australia

**Keywords:** Movement variability, Sport, Skill

## Abstract

Movement variability is defined as the normal variations in motor performance across multiple repetitions of a task. However, the term “movement variability” can mean different things depending on context, and when used by itself does not capture the specifics of what has been investigated. Within sport, complex movements are performed repeatedly under a variety of different constraints (e.g. different situations, presence of defenders, time pressure). Movement variability has implications for sport performance and injury risk management. Given the importance of movement variability, it is important to understand the terms used to measure and describe it. This broad term of “movement variability” does not specify the different types of movement variability that are currently being assessed in the sporting literature. We conducted a scoping review (1) to assess the current terms and definitions used to describe movement variability within sporting tasks and (2) to utilise the results of the review for a proposed framework that distinguishes and defines the different types of movement variability within sporting tasks. To be considered eligible, sources must have assessed a sporting movement or skill and had at least one quantifiable measure of movement variability. A total of 43 peer-reviewed journal article sources were included in the scoping review. A total of 280 terms relating to movement variability terminology were extracted using a data-charting form jointly developed by two reviewers. One source out of 43 (2%) supplied definitions for all types of movement variability discussed. Moreover, 169 of 280 terms (60%) were undefined in the source material. Our proposed theoretical framework explains three types of movement variability: strategic, execution, and outcome. Strategic variability describes the different approaches or methods of movement used to complete a task. Execution variability describes the intentional and unintentional adjustments of the body between repetitions within the same strategy. Outcome variability describes the differences in the result or product of a movement. These types emerged from broader frameworks in motor control and were adapted to fit the movement variability needs in sports literature. By providing specific terms with explicit definitions, our proposed framework can ensure like-to-like comparisons of previous terms used in the literature. The practical goal of this framework is to aid athletes, coaches, and support staff to gain a better understanding of how the different types of movement variability within sporting tasks contribute to performance. The framework may allow training methods to be tailored to optimise the specific aspects of movement variability that contribute to success. This review was retrospectively registered using the Open Science Framework (OSF) Registries (https://osf.io/q73fd).

## Key Points

1. “Movement variability” is a broad term which is often used in the sport literature without a specific definition of the type of movement variability under investigation.
2. A theoretical framework has been proposed to distinguish three types of movement variability: Strategic variability describes the different approaches or methods of movement used to complete a task. Execution variability describes the intentional and unintentional adjustments of the body between repetitions within the same strategy. Outcome variability describes the differences in the result or product of a movement.
3. The terms provided by the proposed framework can increase the specificity of the type of movement variability investigated which can enhance literature comparisons and aid in practical applications.

## Introduction

Human movement is variable. Even highly skilled individuals who consistently achieve the same outcome show movement variability within a goal-oriented action or task [1, 2]. The broad term “movement variability” has been defined as “the normal variations that occur in motor performance across multiple repetitions of a task” [3]. This definition and subsequent research on the topic have led to a change in the view of variability from being noise or an error to be minimised, into an advantageous attribute [4, 5]. Advantages of movement variability include enhanced task performance [4, 6–12] and the spreading of physiological strain [5, 13–19], both of which are discussed in the following sections. This article focuses specifically on within-individual (also referred to as intra-individual or within-subject) movement variability within sporting tasks. This is to say movement variability that is present when a single person performs the same goal-based task across multiple repetitions [20]. Moreover, given that sport research and performance focus primarily on measuring mechanical variables (e.g. kinematics and kinetics) and their results, this review is focused on mechanical variables of movement and movement variability.

### Movement Variability and Performance

Most sporting tasks seek stability and consistency in achieving the desired result (or “goal”) across multiple repetitions [7, 8, 21–23]. Tasks do not specify the movements to achieve this result, thus movement variability allows this goal to be consistently achieved despite potential constraints (e.g. situation, defenders, pressure) [4, 5, 7, 8, 12, 21–26]. Research has shown that elite performers who achieve stable task results are better at changing their movements to meet the different environments or physiological conditions (e.g. fatigue) that can occur within sporting tasks [4, 11, 12, 23, 24]. For example, basketball athletes have been shown to alter their movements within a jumping task to maintain the same jump height even under the constraint of fatigue [25]. Moreover, elite baseball players displayed increased timing variability on their batting swing resulting in more accurate and frequent hits when compared to novice batters [11]. Within baseball pitchers, it has been proposed that reduced movement variability produces greater consistency of ball location, making pitches more predictable and, as a result, easier to hit [27]. Thus, there is a keen interest in high-performance sporting environments to measure and use movement variability to enhance performance.

### Movement Variability and Injury

Movement variability has applications beyond performance such as its effect on physiological strain and stress. James and colleagues [19, 20, 28] proposed the variability-overuse injury hypothesis with variability existing to redistribute stress to other tissues and avoid exceeding physiological capacity. This hypothesis has since been expanded upon by other researchers (e.g. [17, 29]), building on the well-established mechanism of overuse injuries [20, 30–32]. The variability-overuse injury hypothesis also suggests movement variability may be a method to mitigate these injuries [32]. This is particularly relevant for sporting performance where high tissue forces and repetitive actions are commonplace. Variability is a way to redistribute the repeated high forces to different tissues over time [17, 20]. An example of this is a volleyball athlete repeatedly landing from jumps. Low variability in this scenario may result in repeated application of strain to the same tissue, increasing the risk of subsequent tissue breakdown or injury due to overuse [20].

Acute injuries may also occur when excessive movement variability is present [33, 34]. Excessive movement variability may result in risky behaviours or more unstable actions being attempted [33, 34]. Excessively high variability is associated with increased exposure to unexpected or erratic position changes as well as novel and unfamiliar movements [33, 34]. It is theorised that exceeding a certain high limit of variability provides an unfamiliar stimulus and the ability to effectively control movements is exceeded [18]. For example, a sprinter with high step width variability may be more likely to fall or negatively compensate trying to maintain balance [5, 35]. Research has shown that a group of cross-country skiers performing an indoor skiing task began displaying high movement variability with fatigue [33]. This suggests fatigued performers may produce highly irregular and unpredictable movements [33, 34]. These factors are all potential precursors to an acute injury scenario [33, 34]. Together this information suggests a theoretical goldilocks zone of movement variability may exist regarding injuries [5, 14, 34]. This optimal zone would exist between too little variability (overuse injury risk) and too much variability (acute injury risk) and has been proposed to follow an inverted-U shape. [5, 14, 34].

### Types and Analysis of Movement Variability

There are numerous ways to assess and analyse movement variability for any given task [5, 21, 36–40]. This is because variability is pervasive throughout the multiple levels of movement organisation and can occur in many unique ways [4, 5, 15, 23, 34, 41–45]. For example, within the task of basketball shooting, over multiple attempts it is possible to assess variability in: the choice of shot type, the forces applied during each shot release, or the resultant accuracy of each shot, among many more options. These can all be considered movement variability; however, each of these examples represents a different type of movement variability [4, 20, 44].

**Fig. 1:**
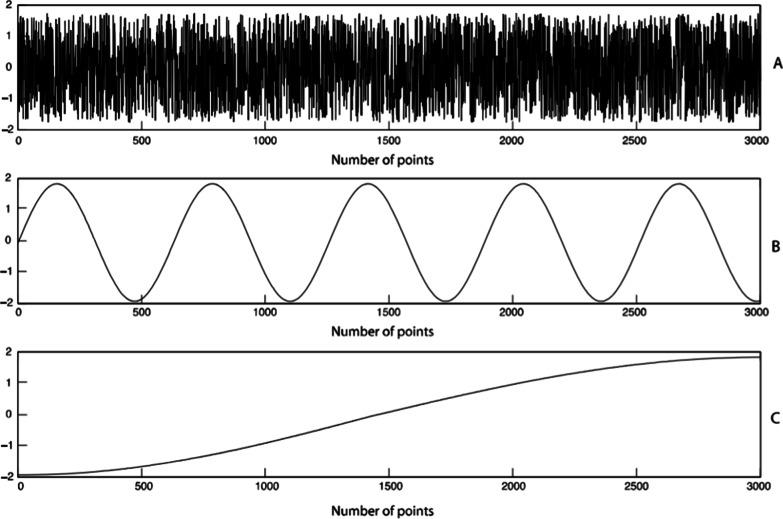
Example of a time series data with the same magnitude of variability (mean = 0, standard deviation = 1) but different structures. Reproduced from Komar et al. [12], with permission

Issues also exist when analysing movement variability, as numerous linear and nonlinear statistical methods can be applied (e.g. [5, 12, 15, 23, 36, 38, 39, 41, 43, 45–48]). Another factor to consider is that variability can be quantified either as a measure of magnitude or a measure of structure [5, 34, 37, 40]. Some quantification techniques, such as nonlinear statistical methods, are sensitive to and consider data structure, whilst other methods do not [3, 36]. Figure 1 provides a visual example of how data can exist with distinctly different data structures despite the same magnitude of variability. Each analysis method has specific considerations as to how it represents movement variability [36–40]. How variability is quantified and what level of movement organisation is measured often reveal what type of movement variability is being investigated [5, 21, 34, 37, 40, 45]. Thus, what is measured and how it is measured are crucial to understand the specifics of the movement variability investigation. These distinctions are necessary because the often-used term “movement variability” is a broad term that does not capture the specifics of what has been investigated.

### Movement Variability Terminology

The term “movement variability” can mean different things depending on the context [4, 5, 9, 12, 16, 17, 42, 43, 49]. Thus, the specific type of movement variability investigated needs to be defined within each context [21]. Failure to define terms may result in similar terms being used interchangeably but with different meanings. The lack of clarity causes difficulties in interpretation and comparison for both readers and researchers. Any misinterpretations from the research could negatively impact practitioners as Preatoni et al. [37] state “the quantification, synthesis, and meaning of movement variability are very important in depicting the athlete’s status and can influence the practical decisions made in sport”.

The following example of anterior cruciate ligament injuries provides evidence of different uses of the same term. The research provided evidence that there is a heightened risk of contralateral anterior cruciate ligament injury post-anterior cruciate ligament reconstruction [50–52]. Some research has taken a systems-based approach to the condition explaining there is low movement variability due to the adoption of changed (i.e. compensatory) movements during the rehabilitation process. Low movement variability in this situation is explained by the compensatory movements, which have turned into a rigid, learned behaviour and in turn a neuromuscular system issue [53, 54]. On the other hand, researchers have also taken a tissue capacity approach to explaining the problem. They have argued there is low movement variability due to a lack of tissue capability in the injured limb, which results in increased reliance on the other limb to accommodate [54–56]. These are both referred to as “movement variability” but represent different levels of movement organisation and provide different information [44]. Distinguishing the type of movement variability within each context is important as practical applications need to be tailored to target specific adaptations [5, 37].

Issues may also occur with the term “movement variability” when comparing research findings. There is the potential for the literature to be compared based on this term even though the comparisons may not be appropriate. Research from Miller [57, 58] and Robins [59, 60] on basketball shooting found both low and high movement variability can result in successful task performance. Each of these studies have used similar tasks and the same term “movement variability”, but they have investigated fundamentally different aspects of movement variability. This can present an issue when studies are compared as the different interpretations of the same term do not provide like-to-like comparisons (e.g. [4, 8]). These examples show how the term “movement variability” needs to be explicitly defined within each specific context to ensure proper interpretation [4, 5, 8, 21, 36–40]. Given the range of interpretations of this term within the sport literature, a review is needed to determine if and how it is being defined in the current sport literature.

## Scoping Review

### Outline

The objectives of this scoping review were twofold: (1) to assess the current terms and definitions used to describe movement variability within sporting tasks and (2) to utilise the results of the review for a proposed framework that distinguishes and defines the different types of movement variability within sporting tasks. Use of this framework may assist in interpreting, contrasting, and applying current research through the synthesis of terms and definitions.

### Methods

We chose a systematic scoping review to map the current state of terms used in the literature. Due to the large and complex nature of the topic, a scoping review was chosen as it avoids appraising study designs and instead summarises the key concepts [61, 62]. The search strategy was framed by the PRISMA-ScR (Preferred Reporting Items for Systematic reviews and Meta-Analyses extension for Scoping Reviews) checklist [63]. This review was registered on 18 November 2020 using the Open Science Framework (OSF) Registries (https://osf.io/q73fd).

The scoping review was conducted on English language, peer-reviewed, and published research journal articles using three searches:

1. “movement variability” AND type AND sport
2. “movement variability” AND term* AND sport
3. “movement variability” AND defin* AND sport

These search terms were selected to provide a representative sample of movement variability in the sporting literature. As discussed in the introduction, movement variability is often referred to as different types [38, 44]. We wished to capture these types, the terms used, and any specific definitions which may be present.

All searches were conducted on 1 January 2022. All years were considered, and searches applied equivalent subjects and related words. A total of five databases were searched; CINAHL Complete, Education Source, MEDLINE Complete, SPORTDiscus, and PubMed. These databases were identified as being relevant to capture multidisciplinary views to movement variability within sporting tasks including what definitions and terms are currently used in the literature. Sources were screened to ensure an appropriate full-text article was available. Sources were eligible if they met the two following eligibility criteria:

1. The source contains at least one quantifiable measure of movement variability
2. The source assesses a sporting movement or skill

These eligibility criteria ensured the scoping review stayed relevant to sporting tasks, and each measure could be objectively identified.

Following screening the remaining eligible information sources were read, and the following data items were extracted:

- The task performed
- The format of collected data (e.g. kinematics, kinetics, outcome, etc.)
- Any explicitly defined terms relating to variability
- Any implicitly defined terms relating to variability
- Any undefined terms relating to variability

Terms were considered explicitly defined if they were associated with a clear definition in the text of the source. Terms were considered implicitly defined by meeting two criteria; no explicit definition was provided; however, an equation or rationale that explained how variability was determined was provided in the source. A data-charting form was jointly developed and used by two reviewers to determine which items to extract. The two reviewers independently charted the data, discussed the results, and continuously updated the data-charting form in an iterative process. Data were critically appraised using descriptive statistics in R (version 3.6.0) [64] to assess the number of defined and undefined terms.

### Results

**Fig. 2:**
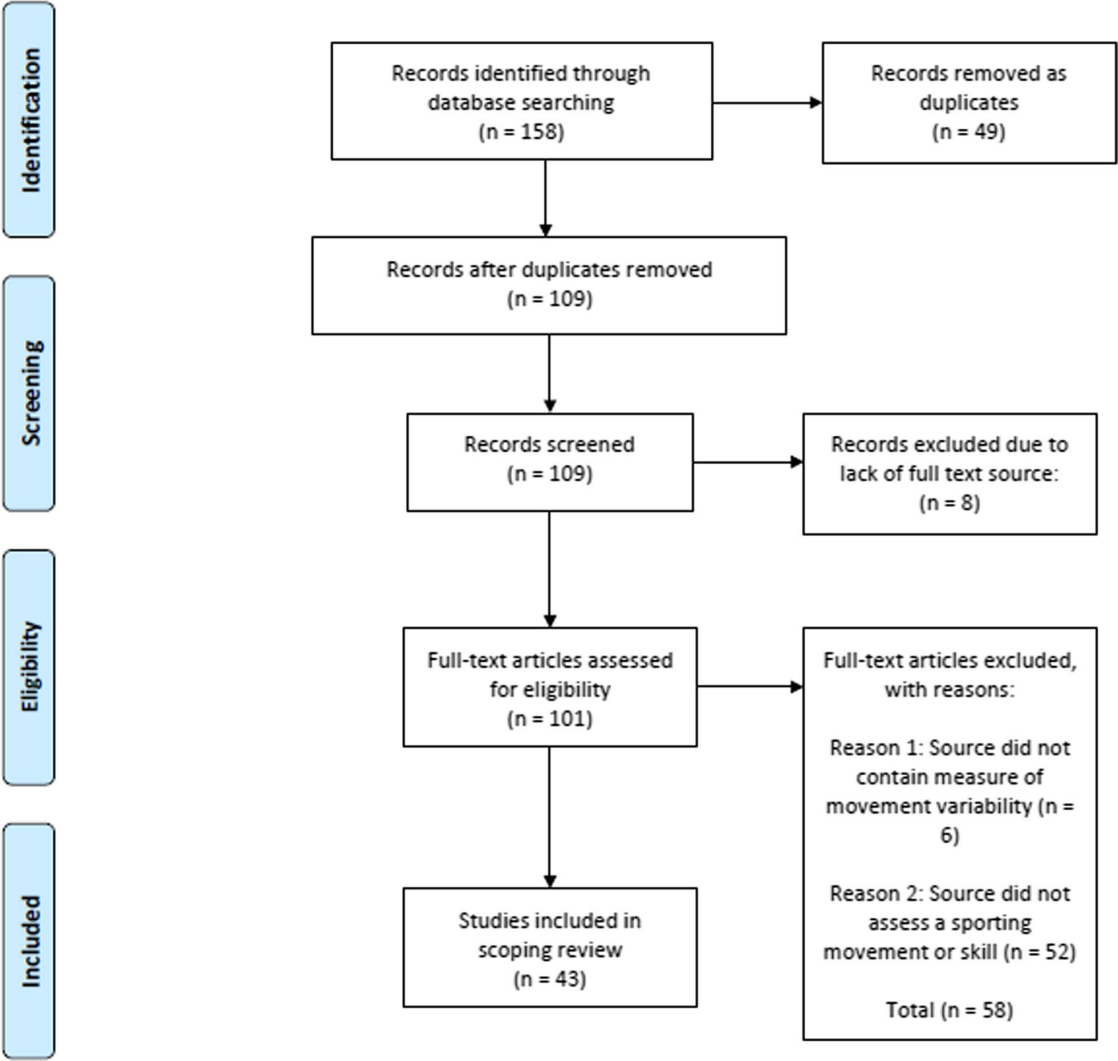
PRISMA diagram showing source inclusion for review. Adapted from: Moher et al. [65]

A total of 158 sources were identified, of which 49 duplicates were removed resulting in 109 sources to be screened. Through initial screening, eight sources were removed as no full text was available (e.g. conference abstract with no relevant or subsequent paper from authors). All remaining 101 full-text sources were screened for eligibility resulting in the removal of an additional 58 sources. This resulted in 43 sources which were included in the review. A PRISMA diagram of this process is shown in Fig. 2, and a table summarising the included sources is presented in Table 1.

**Table 1:** Defined and undefined terms in movement variability literature focused on sporting skills

Study	Task performed	Format of collected data	Explicitly defined terms	Implicitly defined terms (Defined through analysis)	Undefined terms
Aljohani and Kipp [66]	Treadmill continuous long slow distance running	Kinematics, kinetics, and electromyography	–	Continuous relative phase variability Movement patterns Coupling angle variability Vector coding variability	Movement variability Coordination pattern
Bańkosz and Winiarski [67]	Table tennis topspin forehand	Kinematics and outcome	Intra-individual variability Variability Functional changeability Equifinality Random variability	–	Movement variability Functional variability Inter-individual variability Motor variability Movement functionality Specific variability Coordination patterns
Barris et al. [7]	Springboard diving	Kinematics and outcome	Functional movement adaptability Degeneracy	–	Adaptive movement variability Movement pattern variability Functional variability Performance variability Functional movement variability Functional adaptive movement variability Emergent movement form
Bobrownicki et al. [68]	Dart throwing	Kinematics and outcome	–	–	Joint variability Movement variability
Chang et al. [69]	Multiple tasks	Review	–	Joint angle coordination Coordination variability Variability	Movement variability
Chow et al. [70]	Kicking a soccer ball for goal	Kinematics and outcome	–	–	Movement variability Inter-individual variability Movement pattern variability Macro variability Movement cluster variability Behavioural variability Cognitive-motor strategies
Duarte and Reinkensmeyer [71]	Virtual golf club swing	Kinematics	–	–	Kinematic variability
Fargier et al. [72]	Squat vertical jump	Kinematics	Intra-limb variability Inter-limb variability	Segmental coordination types	–
Floria et al. [73]	Countermovement vertical jump	Kinetics	–	–	Multiple-trial variability Jumping variability
García-Pinillos et al. [74]	Continuous treadmill running	Kinematics	–	–	Step variability Spatial and temporal step kinematic variability Gait variability Spatiotemporal variability
Gorman and Maloney [24]	Basketball shooting	Kinematics and outcome	–	–	Movement variability Movement pattern
Grassi et al. [75]	Gymnastics flic-flac	Kinematics	–	–	Spatiotemporal consistency
Guignard et al. [76]	Swimming	Review	Adaptability Stability Flexibility High-order parameters Movement and coordination variability Degeneracy Isofunctionality Coordination patterns	–	Inter-cyclic variability Movement system variability Coordination dynamics Functional variability Inter-cycle variability Movement coordination Performance variability Within-cycle variability Between-cycle variability Inter-individual variability Behavioural variability Inter-cyclic movement variability Inter-arm spatial–temporal coordination Inter-arm coordination Coordination strategies
Hamill et al. [14]	Multiple tasks	Review	Coordinative variability End-point variability Dynamical variability Measurement noise	–	Goal variability
Harrison et al. [77]	Vertical jump	Kinematics	Coordination	Inter-joint coordination patterns	Coordination variability
Haudum et al. [78]	Continuous treadmill running	Kinematics	–	–	Joint coordination variability Functional variability Intervention-induced variability Joint coupling variability Movement variability Within movement variability Acute variability Intralimb inter-joint coupling variability
Hiley et al. [79]	Gymnastics giant swing	Kinematics	Functional variability	–	Kinematic variability Movement variability Timing variability Angle variability
Hodges and Franks [80]	N/A	Review	–	–	Movement variability Coordination pattern Intra-individual variability Within-trial variability Between-trial variability Response variability Within-trial performance variability
Irwin et al. [81]	Gymnastics long swings	Kinematics	–	–	Movement coordination pattern Coordination variability End-point variability Inter-participant variability Movement variability
Komar et al. [82]	Swimming	Kinematics	Inter-limb coordination Neurobiological degeneracy Pluripotentiality Functional coordination	–	Movement variability Movement pattern variability Functional variability Coordination variability
Komar et al. [12]	N/A	Review	Dexterity Variability Intra-trial variability Inter-trial variability Inter-subject variability	–	Adaptability Inter-individual variability
Langdown et al. [83]	Golf	Review	Strategic movement variability Movement variability Functional movement variability Detrimental movement variability Inter-subject variability	–	Coordination patterns Intra-subject variability
Malhotra et al. [84]	Golf swing	Kinematics and outcome	–	–	Movement variability
Marquardt [85]	Golf putt	Kinematics, kinetics, and outcome	–	–	Movement automation
Maurer et al. [86]	Goal-oriented throwing task	Kinematics and outcome	–	Timing/temporal variability	Intrinsic variability Release variability
Middleton et al. [87]	Cricket bowling	Kinematics	–	–	Inter-trial variability Movement variability
Mohammadi et al. [88]	45° side-step cutting	Kinematics and kinetics	Degeneracy Functional variability Coordinative variability Limited variability Structured variability Intrinsic variability Externally imposed variability Flexibility	–	Adaptive movements Practice variability Execution variability Task goal variability Movement pattern Movement solutions Movement coordination repertoire
Orth et al. [89]	Boxing	Kinematics, kinetics, and categorical data	Motor skill Coordination solution changes Control solution changes Task success Functionality Fluency Flexibility Persistency Originality Creativity Exploratory efficiency Coordination switching ratio Control switching ratio	–	Movement variability Exploration movement variability Functional movement variability
Reeve et al. [90]	Landing	Kinematics	–	–	Kinematic variability Intrinsic variability Discrete phase variability
Santos et al. [91]	Small-sided games of Soccer with mixed balls	Outcome	–	Creative movement behaviours Fluency Versatility	Inter-team coordination patterns Movement adaptability Task variability Movement exploration Movement pattern Movement (re)organisation
Sayers [92]	Lawn bowling draw and drive shots	Kinematics and kinetics	–	–	Movement strategies Functional movement variability Movement variability Intra-individual variability Positional variability
Schaefer et al. [93]	Cricket bowling	Kinematics and kinetics	–	–	Movement variability Coordination variability Technique variability
Seifert et al. [94]	Ice climbing	Kinematics and categorical data	Movement variability Degeneracy Multi-stability Meta-stability Inter-limb coordination In-phase mode of coordination Anti-phase mode of coordination Intermediate phase mode of coordination Attunement	–	Functional intra-individual movement variability Inter-limb coordination patterns Movement pattern variability Adaptive movement pattern variability
Slobounov et al. [95]	Springboard diving	Kinematics	–	–	Movement strategies Behavioural flexibility Movement variability Movement patterns Outcome variability Cognitive behavioural strategies
Strongman and Morrison [96]	Review on injury and gait (including running)	Kinematics and electromyography	Stability Rigidity	–	Gait variability Movement variability Joint variability Muscle activation variability Movement patterns Gait patterns
Tanaka and Sekiya [97]	Golf putting	Kinematics, electromyography, and psychological scales	–	Inter-trial variability	Movement variability
Torres [98]	Martial arts jab	Kinematics	–	–	Motor variability Movement variability Movement trajectory variability Variability patterns Micro-movements’ variability
Trounson et al. [99]	Shuttle runs with wearable resistance	Kinematics	Multi-stability Compensation pattern	Movement clusters Angle–angle variability Between-run variability Adaptation strategy Attractor state stability Behavioural meta-stability	Coordination patterns Adaptability Coordinative structure Movement variability Movement system degeneracy Task variability Movement strategies Joint kinematic variability Kinematic variability Movement options Joint angle variability Between-trial variability Functional movement adaptability Movement pattern flexibility Movement states
van Ginneken et al. [100]	Goal-oriented throwing task	Kinematics	–	Trial-to-trial movement variability	Movement variability Trial-to-trial variability
Wang et al. [101]	Running and sprinting	Kinematics	Trial-to-trial variability	Stride length variability Continuous relative phase (CRP) variability Movement variability Cadence variability Intralimb coordination variability Inter-limb coordination variability Angle variability Single joint variability	Running variability Flexibility patterns Movement patterns
Wilson et al. [102]	Triple jump	Kinematics and kinetics	–	Coordination variability Between-trial and within-participant coordination variability	Functional variability Movement variability
Wren et al. [103]	Drop jump, heel touch, and single-leg hops	Kinematics and kinetics	Trial-to-trial variability Trial-to-trial intra-individual variability	Within-subject variability Median variability	Movement patterns Chaotic pattern Movement variability Kinetic variability Kinematic variability
Yang and Scholz [104]	Goal-oriented throwing task	Kinematics and outcome	–	Performance variable variability Movement direction variability Inter-joint coordination Joint configuration variance	Spatial variability Coordination patterns

One source out of 43 (2%) provided definitions for all terms relating to movement variability discussed within the source context. Of the 43 sources investigating sporting tasks, there were 280 terms relating to movement variability. Of these 280 terms, 111 (40%) were defined, while 169 (60%) terms were undefined. From the 111 terms that were defined, 74 (67%) were explicitly defined in the source, while 37 (33%) were implicitly defined. Kinematics were the primary format of data collection, with 34 out of 43 sources (81%) including some measure of kinematics. Outcome results were also investigated in 10 sources (23%) and kinetics in nine sources (21%).

### Discussion

The scoping review provides evidence that a large range of terms are used to describe movement variability within sporting tasks. However, few definitions are provided in relation to each specific context. These results are consistent with prior literature which has concluded that various terms and lack of definitions may be contributing to difficulties in interpretation [4, 5, 8, 17, 36]. The difficulty in interpretation is further supported by Stergiou and Decker [5] who state “much of the controversy that exists in the literature with respect to human movement variability stems from the methodology used”. Similar concerns have been raised by Caballero et al. [36] who claim “the use of so many variables to assess the motor variability have caused problems with the lack of specificity about what variability is”. Without clear definition of these terms in context, it is possible that research may compare the same term despite representing contrasting types of movement variability [4, 8, 17, 57–60].

**Fig. 3:**
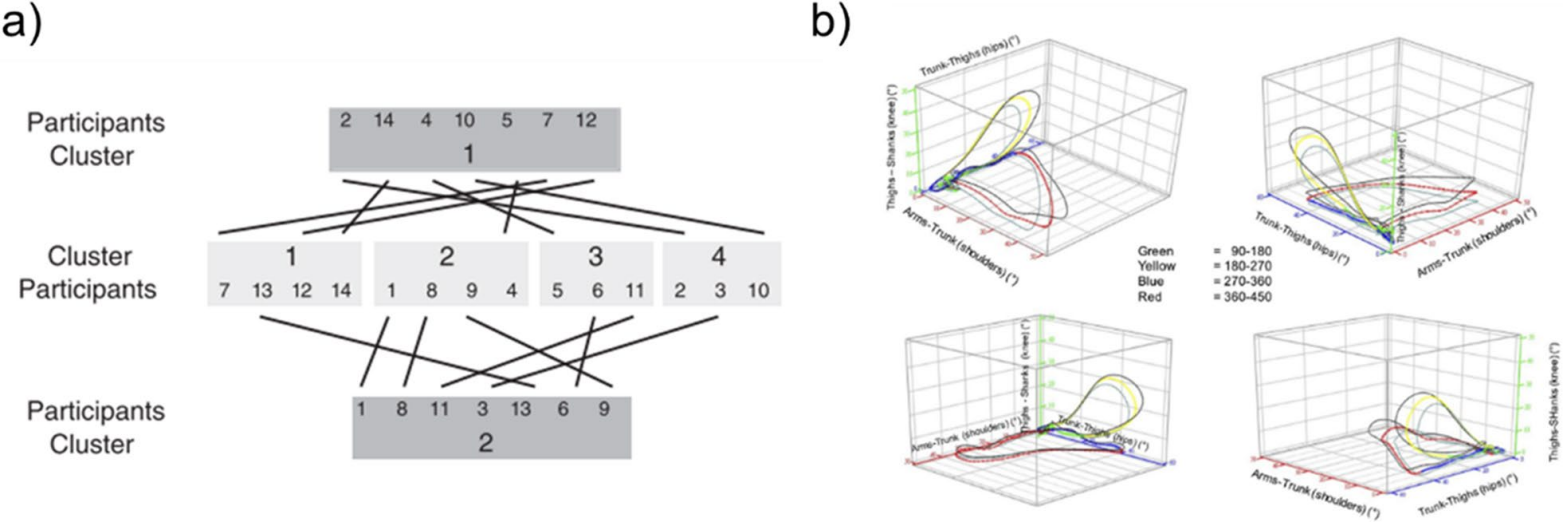
Comparison of the use of the term “coordination variability” across research. **a** Correspondence between clusters’ participants in highly constrained environment (dark grey) and weakly constrained environment (grey). Adapted from Komar et al. [82], with permission. **b** Continuous relative profiles and associated SD of the Trunk-Thigh (hips), Thigh-Shank (knees), and Arm-Trunk (shoulders) plotted simultaneously. *Notes*: Axis: *x* = shoulder, *z* = knees, *y* = hips. Angular position of the gymnasts denoted via colour coding. Adapted from Irwin et al. [81], with permission

We found that the same terms were used across multiple sources but with diverse meanings. The term “coordination variability” is an example of how the same term can be used in two different contexts, be supported by different data analysis approaches, and represent different types of movement variability. “Coordination variability” is a common term both defined and undefined across sources (n = 7). For example, in Komar et al. [82], “coordination variability” is used to distinguish the different clusters of technical solutions shown to perform the task. Based on their technique and performance variables, participants were assigned to a cluster indicating the solution they used (Fig. 3a). In comparison, Irwin et al. [81] used the same term to describe the point-by-point changes in a time series of joint angles over repeated trials of a skill (Fig. 3b). Despite the same term being used, the type of movement variability investigated is different between the studies. Like “movement variability”, the term “coordination variability” used in isolation does not capture the specifics of what has been investigated. The use of the same term to explain different types of movement variability may contribute to a lack of consensus and generalisability within the literature [4, 5, 8–10, 12, 17, 22, 23, 34, 36, 37, 40, 44, 46, 47, 79, 82, 102, 105–110]. Similar issues can arise when placing many terms in front of the word variability. For example, the term “joint variability” is used in multiple sources without definition. Without definition or adequate context, this term may be interpreted as the changes in the position of the joint as it ends a task, the joint used to perform the task, or how the position of the joint is changing during the task.

Results of the scoping review suggest that researchers need to be more specific and explicit in defining what type of movement variability they are investigating. Currently, the state of the literature requires investigation of individual sources to understand how each term was interpreted [21]. The non-specific and non-explicit use of terms is not a new problem, as Newell [111] discussed similar issues within motor control research and application. Newell [111] showed that multiple terms (“coordination”, “control”, and “skill”) were being used interchangeably with different interpretations. This led to the development of a framework that specified each term and explicitly defined them in order to distinguish investigation areas [111]. A similar framework is needed within the movement variability in sport literature; however, sport settings also present specific challenges which need to be considered.

Sporting tasks need to be assessed within ecologically valid domains, such as during competition [22, 24, 112]. Removing sporting tasks from their target domain has been shown to cause individuals to produce different movements despite the same task [24, 113–117]. Understanding how these task solutions change (or do not change) within the target setting and under different conditions is important for skilled performance and injury risk [4, 5, 8, 14, 17, 20, 22, 28, 33, 34, 53]. It is important for practitioners to understand these changes to inform training methods to enhance performance and decrease injury risk [5, 14, 17, 29, 34, 118–122]. Thus, a framework to specify and explicitly define the types of movement variability should consider these challenges to ensure it applies to sporting settings.

## Theoretical Framework for Describing Movement Variability

### Outline

Movement variability is an over-arching complex measure comprised of several different types of variability [4, 7, 9, 14, 18, 21, 23, 38, 39, 41, 43, 45]. The scoping review covered in “Scoping Review” section shows that many terms are used in the literature to describe the distinct types of movement variability; however, they are not well defined. Our framework provides specific terms with explicit definitions to describe the different types of movement variability within sporting tasks. As defined by Crick and Koch [123], “a framework is not a detailed hypothesis or set of hypotheses; rather, it is a suggested point of view for an attack on a scientific problem, often suggesting testable hypotheses”. Rycroft-Malone and Bucknall [124] elaborate on this by stating, “their purpose is in providing a frame of reference, for organising thinking, as a guide for what to focus on, and for interpretation”.

**Fig. 4:**
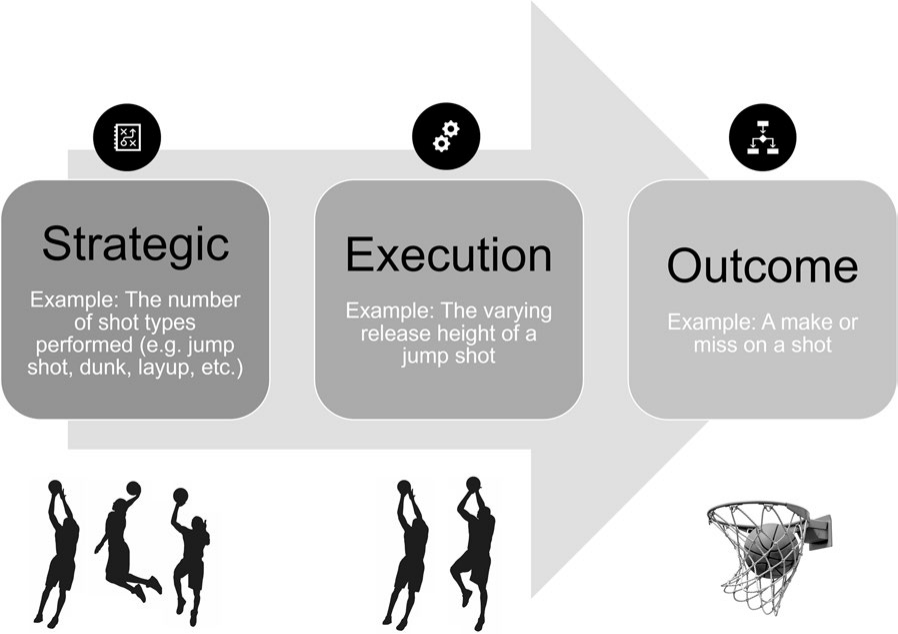
Theoretical framework for describing movement variability with a basketball shot example

Our proposed framework distinguishes three types of movement variability found in the literature: strategic variability, execution variability, and outcome variability. Strategic variability describes the different approaches or methods of movement used to complete a task. Execution variability describes the intentional and unintentional adjustments of the body between repetitions, within the same strategy. Finally, outcome variability describes the differences in the result or product of a movement. A visual representation of this framework is presented in Fig. 4, with definitions of each type of variability explained in the following sections. Moving left to right the framework follows the current understanding of movement action to produce a result. Applying the framework can be summarised as follows: to solve a movement problem an athlete will select a *strategy* from a pool of appropriate strategies that suit the athlete’s constraints, environment, and task. The strategy is then *executed* by moving the body in a certain manner to produce a resultant *outcome.* The terminology in the proposed framework should provide a more specific description of the type of movement variability investigated, and it can also help reduce different interpretations of the same term (see “Types and Analysis of Movement Variability” and “Movement Variability Terminology” section). “A Framework to Describe Movement Variability within Sporting Tasks” section outlines the proposed theoretical framework, and “Application of the Framework: Practical Examples” section provides practical examples to show how the framework can be applied in different settings.

### A Framework to Describe Movement Variability within Sporting Tasks

#### Strategic Variability

Strategic variability describes the different approaches or methods of movement used to complete a task. As defined by Bates [125] “a strategy is a selected musculoskeletal solution for the performance of a motor task. Strategy selection can be voluntary or involuntary”. The strategies available to someone during a task are often based on the relevant environmental constraints, and the individual ability to perform the action needed [23, 125–129]. Strategies exist as task-dependent categorical classifications, and to be considered different, strategies must be qualitatively or quantitatively distinct from one another [130]. The quantity of strategic variability of an individual can then be measured via the number of strategies used to perform the task across repetitions. For example, when observing the outcome of throwing a ball to hit a target, the individual may perform some throws overhand and some throws sidearm; this is an example of strategic variability as the strategy to perform the task has changed across repetitions. Similarly, in soccer when kicking to a teammate, an athlete may kick a lob pass, or a direct pass. These different kick types are different strategies that when performed for the same task show strategic variability. These examples of strategic variability are all qualitatively distinct, but strategies can also be quantitatively distinct from one another.

The determination of strategies is dependent on the research question or application, and different strategies may not always be visually distinct. Other measures and methods may be used to determine the strategies’ categorical classification [131–137]. Clear statements or definitions by the researcher/practitioner of how the strategies are categorised can reduce grey areas between strategies, particularly when strategies may not be as distinctive. An example of this is when multiple strategies reflect two locations on a continuum. Corcos et al. [130] explain this concept using a baseball pitching example, “different variations are likely to be found between a fast ball and a curve ball and these two patterns of variation are probably more understandable as qualitatively different than if lumped together as a function of pitch speed…The two strategies are different in the same sense that the two kinds of pitches are different, and the fact that there are probably movements and pitches that fall between the two extremes does not invalidate the useful notions implicit in creating conceptual categories in the first place”. In sporting literature, strategies within a task have both been defined a priori (before the movement) with pre-determined criteria [15, 18] or a posteriori (after the movement) using data analysis methods to group quantitatively similar performances such as clustering or principal components analysis [131–137]. For example, the term “coordination variability” by Komar et al. [82] discussed in “Discussion” section used a cluster analysis technique on discrete metrics to retrospectively determine performers’ strategies (Fig. 3a). This is an examination of strategic variability according to the proposed framework as retrospectively the movements are quantitatively distinct, despite visually being similar.

#### Execution Variability

Execution variability describes the intentional and unintentional adjustments of the body between repetitions within the same strategy. This relates to the small variations that occur even when trying to complete the exact same movement identically [1, 4, 5, 8]. Bernstein [1] explained this phenomenon as “repetition without repetition”, (i.e. repeating the same task without following the exact same formulaic execution). Execution variability is the most common type of movement variability that is investigated in the current literature. An example of execution variability would be the changes in knee and hip angle coordination, which even when running on a fixed speed treadmill show changes over strides [138, 139]. Unlike strategies which must end in categorical classifications, execution variability can be quantified through many different measurement and analysis techniques (e.g. [4, 5, 9, 12, 16, 17, 21, 42, 43, 49]). The data collected and the analysis method selected to assess execution variability should be specific to the research or application intended. For example, continuous measures such as force–time curves, and discrete measures such as peak force produced by a joint can both be used to measure execution variability. These can be analysed using both linear and nonlinear approaches; for a summary of analysis techniques, refer to the following references [5, 21, 36–39].

**Fig. 5:**
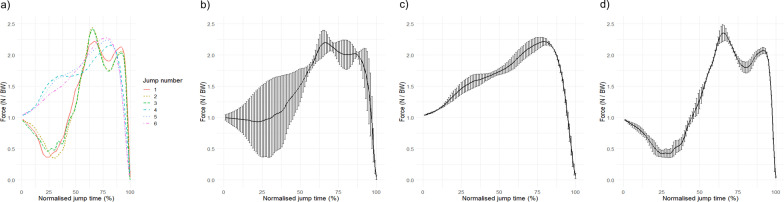
Examples of strategic and execution variability using vertical ground reaction force traces from a vertical jumping task. All forces are normalised to percentage time and expressed relative to body weight (N/BW = Newtons per body weight). (Unpublished data). **a** Six vertical ground reaction force traces from two different movement strategies. Jumps one to three performed with a countermovement and jumps four to six performed with no countermovement. **b** Point-by-point mean and standard deviation of all jumps showing execution variability when jumps are not separated by strategy. **c** Point-by-point mean and standard deviation showing execution variability when jumps using a countermovement strategy are separated for analysis. **d** Point-by-point mean and standard deviation showing execution variability when jumps using a non-countermovement strategy are separated for analysis

Execution variability needs to be quantified within a single strategy. Quantifying execution variability across multiple strategies can produce inflated variability measures. For example, comparing the changes in wrist angle over multiple forehand shots in tennis demonstrates a measure of execution variability. However, combining the analysis of a forehand and a backhand together would provide large amounts of variability due to the different strategies used across repetitions. Comparisons such as this are difficult to interpret as the execution variability is confounded by two strategies. To demonstrate this, Fig. 5a shows six time-normalised vertical ground reaction forces from a vertical jump task (where the individual jumps as high as possible to grab a ball) performed with two distinct movement strategies. Jumps one to three are performed with a countermovement, and jumps four to six are performed with no countermovement. These different movement strategies have qualitatively and quantitatively different ground reaction force traces. Figure 5b provides point by point execution variability (represented by mean and standard deviation) where jumps from both strategies are analysed together. This produces large variability (measured by standard deviation) that provides limited insight and is due mainly to combining different strategies into the same analysis. In contrast, Fig. 5c, d shows the jumps separated by movement strategy prior to assessing execution variability. This example highlights why execution variability should be quantified across repetitions within the same strategy, as comparisons across multiple strategies can provide inflated measures that are difficult to interpret.

#### Outcome Variability

Outcome variability describes the differences in the result or products of movement. An “outcome” is the term used to explain to what was achieved via the movement [1, 23, 37]. Each measure of an outcome needs to relate directly to the task goal but is dependent on the question of interest. What determines the goal of the task, and what outcome measure best exemplifies this? For example, a made basket or missed basket when shooting basketball free throws are examples of outcome measures. Another example of an outcome measure could be the take-off velocity of a golf ball during a tee-off when trying to maximise distance.

Like execution variability, outcome variability can be quantified through many different measurement and analysis techniques [4, 23, 27, 43–45]. The type of data collected and how they are analysed should be specific to the research or application intended. For example, approaches such as success rate (as a percentage) and standard deviation may be used to represent outcome variability in the free throw and golf-ball take-off velocity examples, respectively. Beyond the discrete measures mentioned above the resultant continuous trajectory of an object (e.g. a javelin throw flight path) over repeated executions of a movement may also be used to analyse outcome variability. For a summary of analysis techniques, refer to the following references [5, 21, 36–39].

### Application of the Framework: Practical Examples

#### Basketball Shooting (Open Discrete Task)

The following explains how this framework can be applied to scoring within a basketball game. This situation is representative of an open discrete task according to the definition by Magill [140]. During the game when shooting, a consistent outcome is desired—the ball going through the hoop and registering a goal. The athlete with ball in hand can choose from a pool of finite strategies that they believe will allow them to achieve this goal. These strategies could include a jump shot, lay-up, floater shot, step-back, etc. These strategies are dictated by the performer’s knowledge, skill, and current environmental demands (e.g. defender presence). It is then up to the athlete to select one of the strategies that best fits the current situation to be executed. Consider a scenario where in 10 attempts with the exact same defensive presence and shot location the athlete attempts five jump shots and five floater shots all of which are successful.

In this specific example, *strategic variability* may be the number of options the performer has available to them prior to the shot. Currently, this is the hardest area to quantify and relies on categorical identification of different shot types shown in similar situations. In the above situation, two strategic options are used (jump shot and floater shot). These are identified a priori and categorised as being qualitatively distinct from one another. *Execution variability* is a measure of the intentional and unintentional adjustments of the body between repetitions, within the same strategy (e.g. within all floater shot attempts or within all jump shot attempts). This may include aspects such as wrist angles, knee angles, and vertical ground reaction forces and is dependent on the research question. Finally, the ball going in for a basket or missing may be indicative of *outcome variability*. For this scenario, a successful outcome (scoring a goal) with low outcome variability is ideal and desired.

#### Landing (Closed Discrete Task)

**Fig. 6:**
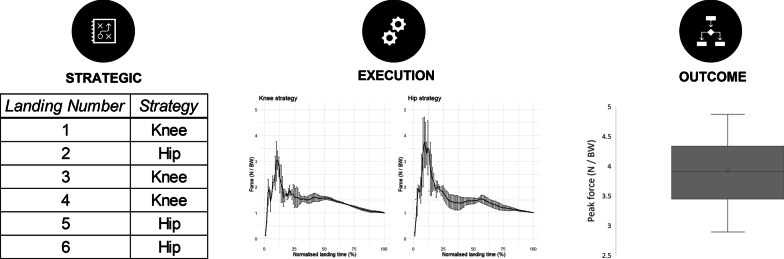
Examples of the three types of movement variability and how they are assessed in a landing task. Strategies are determined by the joint with the greatest energy absorption, with two strategies categorised (knee-dominant landing and hip-dominant landing). Execution variability is represented by the continuous point-by-point standard deviation (SD) of time-normalised vertical ground reaction force traces for all landings within the same strategy. Outcome variability is represented by the SD of the outcome measure (peak force) across all strategies and executions. All forces are normalised to percentage time and expressed relative to body weight (N/BW = Newtons per body weight). (Unpublished data)

In this example, a closed discrete task [140] is chosen whereby an athlete is landing from a jump 20 times. Assume kinetics and kinematics are assessed (such as via 3D motion capture and a force platform), and the outcome of interest is minimising peak ground reaction forces. Applying the framework, outcome variability may be determined by the variability of peak ground reaction force magnitudes. If the researcher or practitioner is interested in determining strategic variability, then they would declare how strategies would be determined. In this example, the criterion for determination of strategy is declared a priori by the joint with the greatest amount of energy absorption. This describes a scenario where the execution variables are used to determine and categorise strategies. This has been done in previous studies (see [15, 18, 141–143]). In the present example, two quantitatively distinct strategies are categorised *knee-dominant landing* or *hip-dominant landing*. Lastly, the variability in the vertical ground reaction force curve within each strategy may be used to assess the execution variability. Figure 6 provides a visual depiction of each type of movement variability mentioned in this example.

Within the above practical example, the joint with the most energy absorbed when landing was used to define a strategy. Thus, multiple landings with a *knee-dominant landing* strategy can have different amounts of energy absorbed and force applied so long as most of the energy absorbed from those landings is at the knee joint. The changes in ground reaction force (e.g. landing one = 3.5 Newtons per body weight, landing three = 4 Newtons per body weight, etc.) can indicate execution variability within the same strategy.

As stated in “Strategic Variability” section, another method to determine quantitatively different strategies is using analysis techniques such as clustering or principal component analysis [131–137]. Applying this approach to the landing waveform data discussed above revealed two distinct clusters in line with current research [143, 144]. These reflected the same categories as the a priori declaration but present another way to determine quantitative distinction and determination of strategies.

#### Running (Continuous Task)

Continuous or cyclical tasks such as walking, running, cycling, and rowing pose unique situations to this framework. Often during these tasks, the execution is itself the outcome, and as such the measure of these types of variability blend [140]. Moreover, depending on the research question or application there may be no strategic variability within a continuous task. However, as explained in “Strategic Variability” section, strategies may also be visually or qualitatively similar despite showing quantitative distinctions [131–137].

Consider the example of running where kinetics and kinematics are collected over 50 stride cycles on a treadmill. There is no set outcome/specific goal to the movement outside of maintaining running velocity and executing the movement itself. If strategic variability is of interest to the researcher or practitioner, then in this example, different strategies may be identified retroactively through the frequency of forefoot, midfoot, or heel-strike ground contact patterns. Each of these types of foot strikes can be determined quantitatively via their distinct vertical ground reaction force traces [145–149]. The execution variability may be identified by assessing the variability of the biomechanics within each of these strategies. For example, this may involve looking at the continuous knee joint angle changes of each step during the forefoot strike strategy.

## Discussion of Theoretical Framework

### How the Framework Fits with Previous Approaches

Utilising a framework to distinguish the types of movement variability is not new [2, 14, 21, 22, 37, 83, 110, 111, 121, 122]. However, the proposed framework in this paper builds upon several earlier frameworks and considers elements that were not addressed previously. This framework provides an explicit consideration of strategic movement variability which is not considered in previous frameworks. This issue was raised by Newell [111] within his paper by stating “the framework proposed does not address directly the issue of strategy although clearly it is an important element of skilled performance”. This paper shares many similar motivations with the paper by Newell [111] who also developed a framework to create distinction between the three terms of “coordination”, “control”, and “skill”. Like the findings in “Scoping Review” section, Newell stated “the distinction between the terms coordination, control and skill is not apparent. Furthermore, perusal of the many academic texts on motor skill learning and motor control reveals a disparity of perspectives on the meaning and significance of these three concepts to the extent there are virtually as many definitions as sources. This inconsistency exists both within a given level of analysis of action (e.g., behavioural) and in a consideration between levels of analysis, such as behavioural and physiological” [111]. By providing a framework that distinguished the terms “coordination”, “control”, and “skill”, Newell outlined how each term represents a unique level of movement organisation [111].

Related frameworks developed by Saltzman and Kelso [121] and Ranganathan and Newell [110] also identified that there is a need to specify terms and distinguish their different interpretations. Each of these frameworks differentiated the result of the movement from the movement itself [110, 121]. Saltzman and Kelso [121] provided descriptive levels of action which separated the outcome of movement, from the body spatial elements involved in the movement, and how these elements were organised. Ranganathan and Newell [110] used a framework to highlight how variability can occur at the level of the task goal (the requirements of the task) and in execution redundancy (the ability to achieve the same task outcome). These descriptions were applied to a sporting example and showed how multiple solutions to the same task can occur despite achieving the same task outcome [110]. Both approaches parallel research by Scholz et al. [122] who applied the terms “essential” and “non-essential” variables to distinguish the variables that influence the task outcome and the variables that do not [150]. They found that to ensure low outcome variability, certain variables needed to display low variability, but other variables were able to show high variability without influencing outcome variability [122, 150]. Each of these approaches enhanced the understanding of skilled movements as each concluded that understanding and analysis at each part of the respective frameworks provided a different and specific understanding of movement [110, 111, 121, 122, 150].

In movement variability research clearly identifying and defining what is being investigated is of high importance. A lack of specificity of terminology and definitions has been previously noted to cause confusion [4, 5, 8, 9, 12, 17, 22, 34, 36, 40, 44, 46, 47, 79, 105, 106, 108–111]. As such, the proposed framework shares an analogous approach to earlier frameworks which aimed to enhance clarity and specificity around what is being investigated and how it influences skilled performance [110, 111, 121, 122, 150]. The proposed framework in this paper aims to address this by providing specific terms with explicit definitions which can be used to imply context across settings. Furthermore, the framework introduced in this paper allows for specific application into sporting environments, which can often present unique challenges such as constantly changing task constraints [22]. Being able to assess skilled movements within the intended performance domain is of key interest in the literature [24, 112–117].

In applying this framework to the scoping review literature, a breakdown of the specific types of movement variability investigated within each study is shown:

- Ten studies provided at least one measure of all three types of movement variability [7, 24, 67–70, 80, 89, 91, 104].
- Four studies provided at least one measure of outcome and execution variability [84, 85, 92, 101].
- 13 studies provided at least one measure of strategic and execution variability [12, 66, 72, 73, 76, 77, 82, 83, 93–95, 98, 99].
- 15 studies investigated only execution variability [14, 74, 75, 78, 79, 81, 86–88, 90, 96, 97, 100, 102, 103].
- One study investigated only outcome variability [71].

By applying the framework, terms that were identified as undefined through the scoping review are now grouped based on the type of movement variability investigated. In doing so, this framework provides a method to specify how the terms are being used, which may create opportunities for more like-to-like comparisons of terms within the literature. Furthermore, being able to distinguish between diverse types of movement variability investigated can provide insights into how movement is organised in sport settings [22]. Application of the framework can be used to help specify and distinguish changes within the different types of movement variability. This can be useful information to guide and help practical applications in sport such as improving sporting performance, mitigating injury risk, and maximising rehabilitation results.

### Practical Considerations

The practical goal of this framework is to aid athletes, coaches, and support staff to gain a better understanding of how the different types of movement variability within sporting tasks contribute to performance. This will allow training methods to be tailored to optimise the specific aspects of movement variability that contribute to success and minimise the others. Ranganathan et al. [22] have stated that “although there are plenty of examples of elite players changing their movement pattern to improve performance or reduce injury, there is little information available on the process of how this reorganisation occurs”. The proposed framework provides a method to help understand how and where this reorganisation is occurring. Furthermore, the proposed framework also aligns with the approach of Ranganathan et al. [22] who identified that in sport settings, changes in movements fall into either explicit, “strategy-like” behaviours or implicit, “synergy-like” behaviours. The strategy-like and synergy-like behaviours align with the proposed strategic and execution types of movement variability, respectively. Distinguishing these types of movement variability is important as expert performers display consistent outcomes despite multiple means of completing a task, i.e. they display both strategic and execution variability [4, 7, 8, 12, 22, 23, 83]. By distinguishing these types from one another, research may be able to yield greater understanding of how expert performers organise their movements when performing tasks in their specific domains [4, 7, 8, 12, 22, 23, 83].

If an individual displays low strategic or execution variability, this lack of adaptability may be exploited by the opposition [4, 8, 13, 24, 27]. For example, a basketball player may have high success with low outcome variability when they drive to the basket with their right hand; however, being forced to their left hand by defenders they have higher outcome variability and thus less success. Defenders aware of this could then heavily guard the right hand forcing more left-hand drives and thus reduced success. Identifying these deficiencies allows for coaches and support staff to implement training methods to develop these abilities [88, 91, 151, 152]. Barris et al. [7] promoted execution variability within the diving training environment and found not only increases in execution variability but also increases in performance consistency post-intervention. This was achieved despite some initial resistance from coaches [7]. Typically, coaches have viewed a successful, low outcome variability as being related to a rigid technical model (low execution and strategic variability) [1, 4, 5, 7, 8, 12, 22, 23, 42, 43, 83, 110, 152]. The proposed framework in this paper may be used as a tool to help show coaches the different types of movement variability and how it may be advantageous to have high or low execution and strategic variability in different settings [4, 5, 7, 8, 12, 23, 152].

A second practical application of the proposed framework is within sports injury and rehabilitation settings [5, 13, 14, 17, 19, 20, 28, 29, 53, 120]. Providing ways to mitigate the risk of injury and re-injury is a key job role for many practitioners. As discussed in “Movement Variability and Injury” section, there is a theoretical relationship between the magnitude of movement variability and overuse injury [14, 17, 19, 28, 29]. Understanding what type of movement variability is changing, and how, offers insights on if the body is adapting (or not adapting) to demands [5, 33, 34, 132]. The ability to conceptualise what type of movement variability is reduced has implications for athlete management and training interventions. Studies have shown that when injured, under fatigue or under increased task demands the amount of strategic and execution variability is reduced [5, 22, 34, 110, 132]. This suggests stress is being applied repeatedly to the same tissues which may result in injury [17, 20, 30–32]. It is also unclear what happens if the task constraints change, and the available strategic and execution variability options are no longer viable. It has been postulated that this may increase the risk of acute injuries [5, 14, 33, 34]. Applying the proposed framework in this paper allows for these, and other theoretical questions to be investigated. For example, it is well established that landing from a jump with limited knee flexion produces a large vertical ground reaction force [143, 153]. This high force is considered a negative by most practitioners, and techniques to reduce these peak vertical ground reaction forces are often taught [142–144]. However, it is unknown if the athlete is better off landing with large ground reaction forces if different strategies are used over time (e.g. hip-dominant strategy and knee-dominant strategy)? Or if the athlete is better off performing a strategy that results in lower ground reaction forces but only using one strategy over time? This is to say, is the athlete better off to have high strategic variability and high force, low strategic variability and low force, or another combination? The proposed framework may provide the specific terminology and understanding to help practitioners to explore this question.

Mitigating the risk of injury and re-injury is a key job role for many practitioners, and understanding how the framework can be applied in rehabilitation settings can result in better short-term and long-term outcomes for athletes. Applying this framework allows practitioners to monitor the different types of movement variability to ensure optimal performance and return-to-play criteria. Within rehabilitation settings, it has been suggested that monitoring movement variability may be a more sensitive marker for return to sport than traditional measures [14, 154]. In research by Seay et al. [154] even runners who were considered recovered from low back pain injuries still showed lower execution variability than those who had never been injured. This suggests a potential re-injury mechanism as a smaller section of tissue is being increasingly stressed in line with the stress-overuse injury hypothesis [14, 17, 19, 20, 28–32]. Targeting this specific type of movement variability with training interventions may enhance execution variability and result in better long-term outcomes for these individuals. Similar findings have occurred in individuals with anterior cruciate ligament reconstructions [53, 88, 155–157]. Stergiou et al. [157] found that those with reconstructed anterior cruciate ligaments displayed less execution variability in the involved limb when walking compared to the non-involved limb [53, 157, 158]. This movement rigidity is suggested to have implications for osteoarthritis and articular cartilage degeneration as the same articulating surfaces are loaded repeatedly over time [5, 159–161]. Thus, applying the framework to understand the types of movement variability and specific interventions for each type may be beneficial to long-term rehabilitation outcomes.

## Limitations

This framework is not without limitations, one of which is that human movement is inherently complex [4, 7, 9, 14, 18, 21, 23, 38, 39, 41, 43, 45]. The focus of this review was on the mechanical variables of movement variability; however, there are more types of movement variability (e.g. neural variability), which were not considered that could explain other important aspects of movement and behaviour [41, 162, 163]. Another limitation is that certain tasks may provide grey areas within the framework where distinguishing each type is difficult. The authors have tried to address this issue within the framework design as discussed in “Strategic Variability” section with reference to the work by Corcos et al. [130]. Limitations also exist within the scoping review process as the literature on movement variability is covered in many different fields [5, 21, 34, 41, 43, 108, 162, 163]. Research outside sporting tasks was excluded, which may have potentially missed some applicable studies, terms, and consensus terminology. Furthermore, this review was conducted in a scoping manner due to the large and complex nature of this topic. This limits results as the focus was narrowed to a subset of the available literature. This subset provides evidence that a more precise and comprehensive systematic review is valid, but the feasibility of such a review must also be considered.

## Conclusion

The scoping review revealed that “movement variability” is a broad term with many different interpretations within the sporting literature. These terms are often not explicitly defined and therefore do not specify what is being investigated. Thus, a theoretical framework is proposed that distinguishes and defines three distinct types of movement variability within sporting tasks: strategic variability, which describes the different approaches or methods of movement used to complete a task; execution variability, which describes the intentional and unintentional adjustments of the body between repetitions within the same strategy, and outcome variability, which describes the differences in the result or product of a movement. By providing specific terms with explicit definitions, the proposed framework can ensure like-to-like comparisons of previous terms used in the literature. By practically applying this framework, athletes, coaches, and support staff can gain a better understanding of how the distinct types of movement variability within sporting tasks contribute to performance. This allows training methods to be tailored to optimise the specific aspects of movement variability that contribute to success.

## Data Availability

The data within this study are secondary data and available through the relevant sources referenced.
